# An Evaluation of Healthcare Safety Culture Among Healthcare Professionals in Secondary and Tertiary Public Hospitals in the Middle East Region

**DOI:** 10.7759/cureus.35299

**Published:** 2023-02-22

**Authors:** Moza A Abdulla, Elmukhtar Habas, Anas Al Halabi, Mohammed Hassan, Farid Sohail, Jameela Alajmi, Hafedh Ghazouani

**Affiliations:** 1 Corporate Quality and Patient Safety, Hamad Medical Corporation, Doha, QAT; 2 Internal Medicine, Hamad General Hospital, Doha, QAT

**Keywords:** hospital survey on patient safety culture (hospsc), psc, patient safety culture, hmc, patient health safety

## Abstract

Background and aim

The provision of quality healthcare is initiated by a culture of patient safety. Understanding the patient safety culture (PSC) is a critical concept for all healthcare workers. We conducted this study to evaluate the PSC understanding among the Hamad Medical Corporation (HMC) staff members. Furthermore, to establish a local (HMC) reference point for providing quality health care based on a culture of patient safety.

Method

A Hospital Patient Safety Culture Survey (HSOPSC) was presented to our health system employees to assess their perceptions and understandings of PSC. The survey was self-administered. STATA Package version 12.0 culture software was used to analyze these data in terms of descriptive, correlational, and multivariate ordinal regression.

Results

This study targeted to survey 6,538 employees in HMC facilities, but only 5,583 responded, resulting in a percentage response rate of 85.4%. Ten facilities achieved 100% participation, and other HMC facilities had response rates ranging from 71.2% to 97.5%. Approximately 88.0% of the responders had direct patient contact. The HSOPSC survey resulted in an overall positive response rate of 62.4%. The dimensions with the highest positive response score were “teamwork within the Unit” followed by “organizational learning/continuous improvement” and “management support for patient safety” with a mean percent positive response (PPR) of 83.1%, 82.0%, and 79.2%, respectively. Conversely, there are three dimensions with the lowest positive response score, including “communication openness,” “staffing,” and “nonpunitive response to errors,” with a mean PPR of 46.6%, 40.1%, and 27.7%, respectively. ANOVA and the student t-test revealed that men (64.3% ± 8.1%), employees with 11-15 years of experience in their specialty (65.8% ± 6.5%), and general hospital type (64.4% ±7.2%), were all significantly associated with differences in the overall perceptions of PSC. According to the study results, there was a moderate correlation between perceptions of PSC at the hospital and the following: Teamwork Across Units (RS= 0.43; p < 0.05), and Frequency of Events Reported (RS= 0.40; p < 0.05.). A regression analysis found that men, workers under 40 years of age, professionals with no direct contact with patients, employees with 11-15 years of experience in their specialty, intensive care staff, and general hospital staff were all significant predictors of overall favorable perceptions of the PSC.

Conclusion

PSC’s understanding of HMC staff is moderate. Furthermore, this is the first study conducted for PSC understanding by the HMC staff in Qatar State. It is eligible to be considered a backbone and reference for new research projects about PSC in Qatari health facilities, if not worldwide.

## Introduction

Employees and leaders must know how to perform their jobs safely, be aware of the risks and hazards present, and be aware of the available control measures if they are to do so in a professional and safe manner. Safety culture assesses “the attitudes, beliefs, and perceptions shared by natural groups as defining norms and values” [1]. Safety culture refers to the shared opinions, beliefs, and attitudes of behavior that guide an organization's approach to promoting health care, productivity, and sustainability. A good safety culture emerges when everyone, regardless of rank or location, has the discipline to obey the rules and the motive to operate as a team. The principles of patient safety culture (PSC) are improving health status and well-being, improving health service delivery, focusing on the differences among those treated, accepting differences, understanding the influence of health services, and how healthcare affects individuals and relatives.

Over the past few decades, the importance of PSC in improving healthcare quality and safety has been raised [2,3]. A PSC is expected to avoid adverse events and promptly fix errors before they cause harm [4]. The PSC components include leadership, evidence-based, teamwork, communication, learning, and patient-centered care subcategories of safety culture [5]. A healthy safety culture in any organization is based on trust, shared ideas about how important safety is, confidence in the effectiveness of preventive measures, and the support of the workforce.

Understanding the quality and safety culture is crucial before questioning the targeted individuals. It was reported that quantitative safety culture assessments offer promise as instruments to improve patient safety [6]. One way to achieve this insight is by using safety cultural assessment tools. The safety culture assessment tools measure organizational conditions which cause patients suffering and undesirable events in healthcare organizations [6].One advantage of measuring safety culture is that it provides tangible indicators of the current situation and how organizations and teams can improve over time [7,8].

The Safety Attitude Questionnaire (SAQ), PSC in Health Care Organizations (PSCHO), and the Hospital Survey on Patient Safety Culture (HOSPSC) are the world's most well-known safety culture assessment measures. The Agency for Healthcare Research and Quality (AHRQ) oversees the HSOPSC, which is a well-known tool with good psychometric properties [9] that is used all over the world. It includes the original English version as well as translations into other languages. The HSOPSC survey assessed PSC at the individual, sectoral, and organizational levels and compared it across sectors and countries [10]. Based on research done in different countries, different institutions and professions have different understandings of patient safety [11-13]. HSOPSC is a tool that has been validated to the point where Cronbach's alpha (a measure of reliability) ranges from 0.7 to 0.8 in data from more than 200 medical practices. Hence, HSOPSC is a reliable method to assess PSC. To our knowledge, only one study has been conducted in Qatar for PSC. The study was done in 2009 and included only 800 single-employee categories (nurses). Therefore, we have conducted this study to evaluate the PSC's understanding and perception among HMC employees, its influencing factors, and its predictors.

## Materials and methods

This study was started on October 25, 2020, for five weeks, utilizing an English and Arabic version of the revised Hospital Survey PSC (HSOPSC) questionnaire [14]. The study included hospital employees, national ambulance services, home care services, and clinical support departments under cover of HMC. Different methods were utilized to facilitate the integration of the initiative into the culture of HMC. Internal access channels, face-to-face meetings, and offering information about the study were offered in the targeted facilities. Employees were informed and encouraged to participate in the study through the webmaster, who serves as the primary point of contact for the whole organization's website (.qa) and is well-positioned to support employees in using the organization's website. In addition, posters and leaflets were distributed throughout the targeted HMC facilities and services to explain the study and its objectives. Hospital and department managers have encouraged their staff to provide feedback via email during their weekly conferences. The seniors received regular updates on survey responses from their departments every week from the study team.

The data was then imported into an Excel spreadsheet and processed by the study team members, including the statistician and the one who collected the data. Data was double-checked and cross-referenced to ensure accuracy by the statistician and the first two authors. Based on the quantified results of the data collection, a safety culture matrix was created to provide a comprehensive overview of data safety.

Instrument (HSOPSC Survey)

The HSOPSC contains 12 dimensions that have 42 items (teamwork within units [four items], supervisor/manager expectations and actions to promote patient safety [four items], supervisor organizational learning - continuous improvement [three items], and management support for patient safety [three items], supervisor cross-departmental teamwork [four items], staffing [four items], handoffs and transitions [four items], non-punitive response to failure [three items], feedback and communication about the failure [three items], and openness to communication [four items]).

A five-point Likert scale was used for each question, indicating whether respondents agreed or disagreed, including a neutral category (1=strongly disagree, 2=disagree, 3=neither, 4=agree, 5 =strongly agree). A positive response was considered when the score was ≥ 4 on a five-Likert scale and considered negative when the score was < 4. Each Likert scale item score is converted to percentage or frequency and is scored on 100, which is the best way to explore the data. Hence, the scores received the following values: the percent of extreme disagreements was 0%, moderated disagreement replies 25%, neutral answers 50%, permeate agreeable responses 75%, and highly agreeable answers 100%. Some answers were based on frequency (never to always). The tool had 59.5% positive and 40.5% negative questions. The survey also included two questions that asked respondents to provide an overall grade of PSC for their work area (A = excellent, B = very good, C = acceptable, D = poor, E = failing) (four items) [15] and the number of reported events they have reported over the past 12 months (three items) [16]. The HOSPSC 2.0 survey instrument had a reliability rating of 0.8 [17]. HOSPSC has been tested and validated in more than 62 studies conducted in 29 countries [18]. The dimensions of the HSOPSC are shown in Table 1, and its properties are shown in Table 2. The set of questionnaires is available on the AHRQ website (www.ahrq.gov/qual/hospculture).

**Table 1:** Agency of Healthcare Research and Quality (AHRQ) patient safety culture composites

Composite	Definition
Communication openness	staff freely speak up if they see something that may negatively affect a patient and feel free to question those with more authority
Feedback and communication about error	staff are informed about errors that happen, are given feedback about changes implemented, and discuss ways to prevent errors
The frequency of events reported	mistakes of the following types are reported: errors caught and corrected before affecting the patient; no potential to harm the patient; and errors that could harm the patient but do not
Handoffs and transitions	important patient care information is transferred across hospital units and during shift changes
Management support for patient safety	hospital management provides a work climate that promotes patient safety and shows that patient safety is a top priority
Non-punitive response to the error	staff feel that their mistakes and event reports are not held against them and that mistakes are not kept in their personnel file
Organizational learning—continuous improvement	mistakes have led to positive changes and changes evaluated for effectiveness
Overall perceptions of patient safety	procedures and systems are good at preventing errors, and there is a lack of patient safety problems
Staffing	there are enough staff to handle the workload, and work hours are appropriate to provide the best care for patients
Supervisor/manager expectations and actions promoting patient safety	supervisors/managers consider staff suggestions for improving patient safety, praise staff for following patient safety procedures, and do not overlook patient safety problems
Teamwork across units	hospital units cooperate and coordinate with one another to provide the best care for patients
Teamwork within units	staff support each other, treat each other with respect, and work together as a team

**Table 2:** Properties of the survey on patient safety culture

No Composites of patient safety culture	Item			Scale
Unit/Department level				
1. Teamwork Within Units		4		5 points Likert (strongly disagree to strongly agree)
2. Supervisor/Manager Expectations & Actions Promoting Patient Safety		4		5 points Likert (strongly disagree to strongly agree)
3. Organizational Learning—Continuous Improvement		3		5 points Likert (strongly disagree to strongly agree)
7. Communication Openness		3		5 points Likert (never to always)
6. Feedback & Communication About Error		3		5 points Likert (never to always)
12. Nonpunitive Response to Errors		3		5 points Likert (strongly agree to strongly disagree)
10. Staffing		4		5 points Likert (strongly disagree to strongly agree)
The aspect of the hospital				
4. Management Support for Patient Safety		3		5 points Likert (strongly disagree to strongly agree)
9. Teamwork Across Units		4		5 points Likert (strongly disagree to strongly agree)
11. Handoffs & Transitions		4		5 points Likert strongly disagree to strongly agree)
Resulting variables				
8. Frequency of Events Reported			3	5 points Likert (never to always)
5. Overall Perceptions of Patient Safety			4	5 points Likert (strongly disagree to strongly agree)

Measures

Benchmarking identifies optimum care practices. Quality measure benchmarking helps professionals improve results. Using the quality measures to find research opportunities that increase professional awareness and guide future best practices by assessing quality measure variance. Quality benchmarks can monitor the improvement. Measures consisted of identifying appropriate outcomes and agreeing on metrics and targets (the output level). Outcome measurement is achieved by identifying the variables needed for measurement and applying them to show whether the outcome has been affected or not. The main outcome of this study is HMC staff PSC understanding.

Outcomes

The perception of the overall HOSPSC means the score was chosen as an outcome-dependent variable. The mean patient safety perception scores were categorized into positive and negative perceptions (≥75.0% and <75%, respectively).

Model estimation

This study used a multiple regression model to predict the relationship between the outcome variable (the overall mean score perception of the PSC), demographic factors, work-related factors, and 12 dimensions of PSC. A correlation coefficient (R2) of 81.6% indicated that the model accurately represented and fitted the data.

Study explanatory variables 

The facility includes general or specialty hospitals, clinical support, and corporate services. The individual-level independent variables were entered as categorical variables encompassing 12 PSC dimensions. Age (<40 years, 40-55 years, ≥ 55 years), sex (male, female), working unit (emergency, intensive care, medicine, obstetrics, out-patient department, pediatrics, pharmacy, psychiatry/mental health, rehabilitation, and surgery), work experience (1-5 years, 6-10 years, 11-15 years, ≥16 years) were used. Furthermore, current specialty experience (1-5 years, 6-10 years, 11-15 years, ≥16 years), patient contact (yes or no), and professional position (HMC administration, paramedic, occupational therapist, physician, pharmacist, physical therapist, occupational or speech therapist, registered nurse and midwife, technician, and others) were all used. The mean weekly working time is divided into three categories: < 40 hours, 40 to 59 hours, and ≥ 60 hours were also entered.

Ethical approval 

The Quality and Safety Department of Hamad Medical Corporation and the Medical Research Center collaborated in conducting cultural surveys starting January 1, 2012. No ethical approval or consent was required for the patient safety of the SOPS® in hospitals. Each biennial report is published, and the agreement is automatically renewed every two additional years.

Statistical consideration and data analysis

A cross-sectional analysis was performed using descriptive statistics such as means, standard deviations, frequencies, and percentages. Pearson and Fisher exact and chi-square tests were performed to assess correlations between groups. A review was conducted to identify inconsistencies in the questionnaires. Samples were analyzed using the Shapiro-Wilk test. The p-value for this sample is 0.004, which is normal.

The answers were divided into four categories: negative, neutral, positive, and non-responsive. When reflecting on positive responses, HSOPSC included positive and negative dimensions. The negative response dimensions are reversed positive scores. For example, for the item “The staff feels like their mistakes are held against them,” 67.9% replied with negative responses, which reversed to 32.1% percent positive responses (PPRs).

We established total scores by calculating the percentage of positive responses (PPR) to the safety culture questions. A PPR score greater than 75% is considered a “strong” safety culture score. On the other hand, a PPR score between 50 and 75% is regarded as a “moderate” safety culture score, and if the score is less than 50%, it is considered a “weak” safety culture score.

Pearson's correlation analysis was used to analyze the correlation between the different dimensions of the PSC (independent variables) and overall mean perceptions of HSOPSC (dependent variable). Further, we assessed differences in overall means of HSOPSC perception by demographic and work category using Student's t-test and one-way F-test ANOVA. Multiple regression identified HSOPSC perception predictors using overall patient safety perception as the outcome of demographic and work variables. The exploratory data analysis and descriptive statistics were conducted using STATA Package version 12.0 (Stata CorpCohen'sge Station, TX, USA). A p-value of ≤ 0.05 is considered statistically significant.

Sample size and sampling procedures

**Figure 1:**
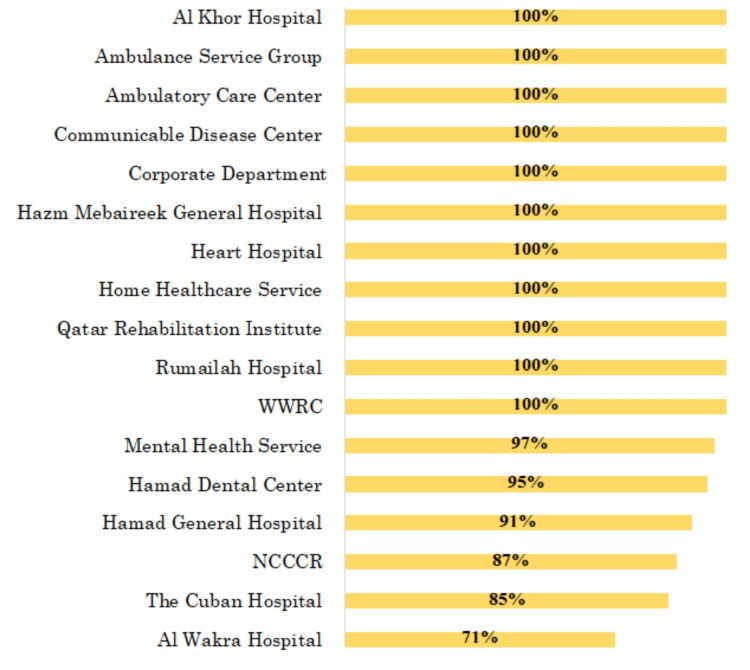
Total survey responses from individual HMC Hospitals and Services

The data were collected from nine hospitals, three community hospitals, an ambulance medical service, a home care service, and support services governed by HMC. A participant's power of 0.8 values and Cohen's linear regression were used for the sample size calculation. The calculated required sample size must composite a minimum of 1,829 participants [19]. Six thousand five hundred thirty-eight people met the study criteria. Only 5,583 participated, with a response rate of 85.4%. The participants’ response rates from the included HMC hospitals are shown in Figure 1.

## Results

Characteristics of participants

There were 5,583 respondents, 64.2% of whom were male. Direct patient contact was reported in 88% of the responders (5,243). The respondents who answer the demographic and work-related characteristics were 5245 employees. The participants’ mean age was 47.6 years, and 30% were aged between 30 and 50 years. They were 2.5% pharmacists, 2.9% physiotherapists and occupational therapists, and 2% laboratory technicians. There were 13.7% of participants worked in outpatient departments. Participants who worked for 1-5 years made up 43.1% of the total, while those who worked for 6-10 years made up 28.1% of the responders (4,477 employees). Respondents who worked <60 hours/week were 92% of those who responded (5,264). Of the HMC facilities included, 82.5% were hospitals, and 13.4% were not hospitals (clinical support and corporate). The demographics and work characteristics of the participants are presented in Table 3.

**Table 3:** The demographics and work characteristics of the participants Others* (nursing assistants, physical therapists, audiologists, occupational therapists, speech pathologists, dieticians, respiratory therapists, occupational, speech therapist, electrocardiogram, lab, radiology technicians, sociologist), number of responders (N), Clinical Support services * (clinical information system, nursing informatics, quality, and patient safety)

Variables	N	%
Gender (N=5,245)		
Male	3,379	64.2%
Female	1,885	35.8%
Age (N=5,245)		
Under 40	3,356	64.0%
40 -55	1,626	31.0%
55 and above	263	5.0%
Position/profession (N=5,245)		
HMC Administration	215	4.1%
Management Ambulance Paramedic	282	5.4%
Staff Physician	241	4.6%
Pharmacist	131	2.5%
Physical, Occupational, or Speech Therapist	150	2.9%
Registered Nurses and midwives	3,152	60.1%
Technician (e.g., EKG, Lab, Radiology)	106	2.0%
Others*	968	18.5%
Interaction with Patient (N=5,243)		
I typically do not have direct interaction or contact with patients	631	12.0%
Yes, I typically have direct interaction or contact with patients	4,612	88.0%
Working unit (N=3,336)		
Emergency	320	9.6%
Intensive care (any type)	409	12.3%
Laboratory and Radiology	166	5.0%
Medicine (non-surgical)	384	11.5%
Obstetrics	361	10.8%
Out-patient department	457	13.7%
Pediatrics	226	6.8%
Pharmacy	155	4.6%
Psychiatry/mental health	173	5.2%
Rehabilitation (including physical therapy)	309	9.3%
Surgery	376	11.3%
Work Experience (N=5,245)		
Less than 1 Year	315	6.0%
1 to 5 Years	2,265	43.1%
6 to 10 Years	1,288	24.5%
11 to 15 Years	702	13.3%
16 Years or More	675	12.8%
Tenure with Current Specialty or Profession (N=4,477)		
Less than 1 Year	119	2.7%
1 to 5 Years	853	19.1%
6 to 10 Years	1,259	28.1%
11 to 15 Years	1013	22.6%
16 Years or More	1233	27.5%
Hours Worked Per Week (N=5,264)		
<40 hours per week	303	5.7%
40 to 59 hours per week	4,567	87.0%
60 hours per week or More	375	7.1%
Types of Facilities		
General Medical & Surgical Hospitals	2,292	43.7%
Specialty Hospitals	2,039	38.8%
Clinical Support services *	698	13.3%
Corporate Services	216	4.2%

PSC items and the level of PSC dimensions 

Among the 42 items of the 12 PSC dimensions, 13 had positive responses above 75.0%, while 11 had positive responses below 50.0%. Table 2 shows that the highest positive response percentage item was for "We are actively doing things to improve patient safety" (92.1%), while item "Please give your work area/unit in this hospital an overall grade on patient safety" had the lowest positive response (16.2%). 

Considering all 12 dimensions, the overall mean of positive perception of PSC by HMC healthcare workers was 62.4%, ranging between 61.5% and 76.4%. However, each of the 12 dimensions had a mean PPR of 27.7% and 83.1%. Three dimensions achieved a mean >75.0%: “teamwork within the unit” (83.1%), “organizational learning/continuous improvement” (82.0%), and “management support for patient safety” (79.2%). Five dimensions had a mean PPR between 50.0% and 75.0% (“the frequency of events reported” [70.8%], “feedback and communication about errors” [70.1%], “supervisor/manager expectations and actions promoting patient safety” [68.1%], “overall perceptions of patient safety” [62.7%], “teamwork across units” [67.1%], and “handoffs and transitions” [59.2%]). The mean PPR of “communication openness,” “staffing,” and “no punish response error” dimensions were (46.6%), (40.1%), and (27.2%), respectively. Table 4 demonstrates the PSC dimensions’ mean PPR. Patient safety grade and number of events reported.

**Table 4:** Mean percent positive response (Mean PPR) of each dimension and each item's percent positive response (PPR) of HMC healthcare workers' perception of patient safety culture. negatively worded item (R).

Dimensions label	Mean PPR of the items in each dimension	Items	Each item PPR
Unit/ Department level			
1. Teamwork Within Units	83.1%	A1. People support one another in this unit.	87.2%
		A3. When much work needs to be done quickly, we work together as a team to get the job done.	85.7%
		A4. In this unit, people treat each other with respect.	85.9%
		A11. When one area in this unit gets bustling, others help out.	73.6%
2. Supervisor/Manager Expectations & Actions Promoting Patient Safety	68.1%	B1. My supervisor/manager says a good word when they see a job done according to established patient safety procedures.	76.3%
		B2. My supervisor/manager seriously considers staff suggestions for improving patient safety.	74.2%
		B3. Whenever pressure builds up, my supervisor/manager wants us to work faster, even if it means taking shortcuts. (negatively worded)	52.4%
		B4. My supervisor/manager overlooks patient safety problems that happen over and over. (negatively worded)	69.3%
3. Organizational Learning—Continuous Improvement	82.0%	A6. We are actively doing things to improve patient safety.	92.1%
		A9. Mistakes have led to positive changes here.	66.8%
		A13. After we make changes to improve patient safety, we evaluate their effectiveness.	87.1%
7. Communication Openness	46.6%	C1. We are given feedback about changes put into place based on event reports.	62.0%
		C3. We are informed about errors that happen in this unit.	39.0%
		C5. In this unit, we discuss ways to prevent errors from happening again.	39.0%
6. Feedback & Communication About Error	70.1%	C1. We are given feedback about changes put into place based on event reports.	59.7%
		C3. We are informed about errors that happen in this unit.	73.3%
		C5. In this unit, we discuss ways to prevent errors from happening again.	77.5%
12. Nonpunitive Response to Errors	27.7%	A8. The staff feels like their mistakes are held against them. (R)	32.1%
		A12. When an event is reported, it feels like the person is being written up, not the problem. (R)	34.2%
		A16. Staff worries that mistakes they make are kept in their personnel file. (R)	28.3%
		E1. Please give your work area/unit in this hospital an overall grade on patient safety.	16.2%
10. Staffing	40.1%	A2. We have enough staff to handle the workload.	55.2%
		A5. Staff in this unit work longer hours than is best for patient care. (R)	37.1%
		A7. We use more agency/temporary staff than is best for patient care.	52.1%
		A14. We work in "crisis mode," trying to do too much too quickly. (R)	19.3%
The aspect of the hospital			
4. Management Support for Patient Safety	79.2%	F1. Hospital management provides a work climate that promotes patient safety.	86.2%
		F8. The actions of hospital management show that patient safety is a top priority.	88.7%
		F9. Hospital management seems interested in-patient safety only after an adverse event happens.	62.9%
9. Teamwork Across Units	67.1%	F4. There is good cooperation among hospital units that need to work together. (R)	58.4%
		F10. Hospital units work well together to provide the best care for patients.	78.1%
		F2. Hospital units need to coordinate better with each other.	43.6%
		F6. It is often unpleasant to work with staff from other hospital units.	88.2%
11. Handoffs & Transitions	59.2%	F3. Things "fall between the cracks" when transferring patients from one unit to another.	57.5%
		F5. Important patient care information is often lost during shift changes.	70.2%
		F7. Problems often occur in the exchange of information across hospital units.	44.8%
		F11. Shift changes are problematic for patients in this hospital.	64.1%
Resulting variables			
8. Frequency of Events Reported	70.8%	D1. When a mistake is made but is caught and corrected before affecting the patient, how often is this reported?	70.4%
		D2. When a mistake is made but has no potential to harm the patient, how often is this reported?	68.3%
		D3. When a mistake is made that could harm the patient but does not, how often is this reported?	73.7%
5. Overall Perceptions of Patient Safety	62.7%	A15. Patient safety is never sacrificed to get more work done.	32.3%
		A18. Our procedures and systems are good at preventing errors from happening.	77.2%
		A10. It is just by chance that more serious mistakes don't happen around here.	56.5%
		A17. We have patient safety problems in this unit.	85.1%

**Figure 2:**
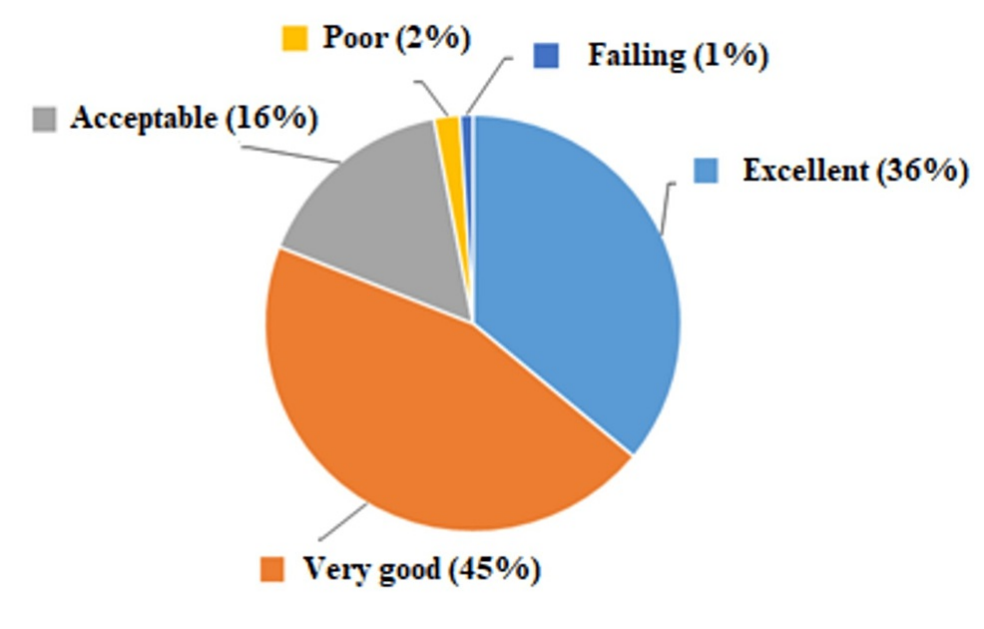
Percentage of respondents' workplace score on overall Patient Safety Grade

Figure 2 shows that 81.0% of respondents graded patient safety as excellent or very good, 16.0% graded it as acceptable, 2.0% graded it as poor, and only 1.0% had a failing response.

**Figure 3:**
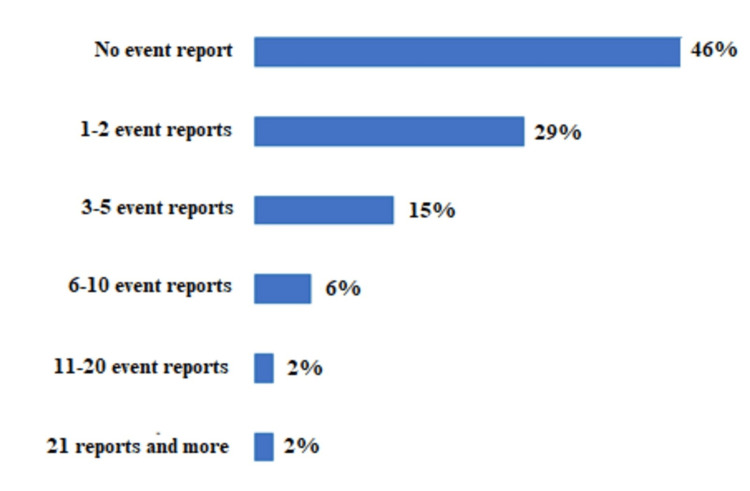
Percentage of respondents reporting events in the past 12 months

A year before starting the survey, 46.0% of the participants reported no patient safety incidents, 29.0% reported one to two incidents, and 25.0% reported more than two incidents (Figure 3).

Correlations and differences in HSOPSC perceptions level

ANOVA and the student t-test analysis revealed that males (64.3% ± 8.1%) had the highest PPR mean scores. Participants with 11-15 years of experience in the same specialty (65.8% ± 6.5%) had significantly higher PPR scores, followed by the general hospital (64.4% ± 7.2%) with a significantly higher mean PPR score in a type of facility group. These categories had a significant impact on the overall mean PSC perception score. There was no significant difference between the PSC score and the age group, profession position, work experience, sex, patient interaction, weekly work hours, and unit of service (p-value > 0.05). Table 5 summarizes the mean PPS by categorical variables.

**Table 5:** Mean percent positive score by categorical variables ^*^t-test (Student T-test) was used as a statistical test, ^**^variance analysis F test was used as a statistical test, a significant difference (p < 0.05)

Variables	Count	Mean percent positive score	The standard deviation of the percent positive score	P-value
Gender				
Male	3,379	64.3%	8.1%	<0.001^*^
Female	1,885	62.7%	7.5%	
Age				
Under 40	3,356	63.1%	8.3%	0.412^**^
40 -55	1,626	61.5%	8.5%	
55 and above	263	64.9%	6.6%	
Current position/role				
HMC Administration	215	61.4%	8.8%	0.064^**^
Ambulance Paramedic	282	63.5%	8.5%	
Staff Physician	241	63.7%	7.2%	
Pharmacist	131	62.4%	6.1%	
Physical, Occupational, or Speech Therapist	150	63.5%	6.4%	
Registered Nurses and midwives	3,152	65.8%	6.4%	
Technician (e.g., EKG, Lab, Radiology)	106	62.0%	6.5%	
Others	968	62.3%	5.7%	
Interaction with Patient				
I typically do not have direct interaction or contact with patients	631	64.4%	8.2%	0.225^*^
Yes, I typically have direct interaction or contact with patients	4,612	62.2%	7.5%	
Working unit				
Emergency department	320	62.9%	6.1%	0.056^**^
Intensive care unit(any type)	409	62.3%	6.4%	
Laboratory and Laboratory	166	60.2%	6.8%	
Medicine (non-surgical)	384	64.3%	5.7%	
Obstetrics	361	62.1%	5.2%	
Out-patient department	457	63.7%	5.1%	
Pediatrics	226	63.2%	5.5%	
Pharmacy	155	62.8%	7.3%	
Psychiatry/mental health	173	63.4%	7.2%	
Rehabilitation(including physical therapy)	309	61.3%	7.1%	
Surgery	376	63.5%	7.2%	
Work Experience				
Less than 1 Year	315	65.3%	8.5%	0.248^**^
1 to 5 Years	2,265	62.7%	6.3%	
6 to 10 Years	1,288	62.3%	6.3%	
11 to 15 Years	702	62.6%	6.2%	
16 Years or More	675	61.1%	5.5%	
Tenure with Current Specialty or Profession				
Less than 1 Year	119	61.0%	6.3%	<0.005^**^
1 to 5 Years	853	64.4%	6.5%	
6 to 10 Years	1,259	62.2%	7.4%	
11 to 15 Years	1,013	65.8%	6.5%	
16 Years or More	1,233	61.7%	5.4%	
Hours Worked Per Week (Hours)				
<40 hours per week	303	64.3%	8.4%	0.485^**^
40 to 59 hours per week	4,567	62.3%	8.7%	
60 hours per week or More	375	60.1%	5.0%	
Facility type				
General Medical & Surgical Hospitals	2,292	64.4%	7.2%	<0.05^**^
Specialty Hospitals	2,039	63.8%	8.3%	
Clinical Support services	698	61.9%	7.5%	
Corporate Services	216	60.4%	6.6%	

A significant correlation (p < 0.05) exists between HOSPSC perception mean score level and patient safety culture dimensions, except for the five dimensions listed above - “organizational learning/continuous improvement,” “open communication,” “non-punitive response to errors,” “transfers and transitions,” and “overall perception of patient safety.” The Pearson correlation results are summarized in Table 6.

**Table 6:** Pearson correlation matrix between patient safety culture dimensions and overall perception of Hospital survey on patient safety culture (HSOPSC) Level of significance <0.05 (P-value*), Not significant (NS), Spearman's rank correlation coefficient (RS)

Patient safety culture dimensions	Absolute values of the Pearson Correlation Coefficients (RS)	P-value*	Strength of the correlation (power)
Teamwork Within Units	RS = 0.21	<0.05	Low correlation
Supervisor/Manager Expectations & Actions Promoting Patient Safety	RS = 0.21	<0.05	Low correlation
Organizational Learning—Continuous Improvement	RS = 0.02	NS	Very low correlation
Communication Openness	RS = 0.06	NS	Very low correlation
Feedback & Communication About Error	RS = 0.14	<0.05	Very low correlation
Nonpunitive Response to Errors	RS = 0.04	NS	Very low correlation
Staffing	RS = 0.24	<0.05	Low correlation
Management Support for Patient Safety	RS = 0.28	<0.05	Low correlation
Teamwork Across Units	RS = 0.43	<0.01	Moderate correlation
Handoffs & Transitions	RS = 0.01	NS	Very low correlation
Frequency of Events Reported	RS = 0.40	<0.01	Moderate correlation
Overall Perceptions of Patient Safety	RS = 0.03	NS	Very low correlation

Compared to the overall perception of the HOSPSC results mean score, power correlations between the 12 HSOPSC dimensions showed weak correlation power (0.01 to 0.43). As found in the study, the best correlations with the overall safety culture perception mean score among healthcare professionals were moderate in “Teamwork Across Units” (RS=0.43; p < 0.05) and “Frequency of Events Reported” (RS=0.40; p < 0.05). The scale used is presented in Table 7.

**Table 7:** Pearson’s correlation coefficient (RS) interpretation

The scale of the correlation coefficient	Interpretation
0 < RS ≤ 0.19	Very low correlation
0.2 ≤ RS ≤ 0.39	low correlation
0.4 ≤ RS ≤ 0.59	Moderate correlation
0.6 ≤ RS ≤ 0.79	Very high correlation
0.8 ≤ RS ≤ 1.0	High correlation

Predictors of Staff Overall Perceptions of HSOPSC

Table 8 summarizes the results of a multiple regression analysis on variables predicting perceptions of HOSPSC among HMC employees. Predictors of participants' perceptions of HSOPSC were as follows. Males had a 17.0% (OR=1.17: p < 0.05) higher positive PSC score than females. Employees under 40 years of age were 68% (OR=1.68: p < 0.05) more likely to have positive perceptions. Professionals, who do not have direct contact with patients, evaluated their safety culture 1.16 times positive (OR =1.16: p < 0.05), and paramedics were 8.0% (OR=0.92: p < 0.05) less likely to have a positive perception of the PSC than the rest of the staff hospital. Employees had 11-15 years of experience in their work specialty were 1.38 times more likely (OR=1.38: p < 0.05) to have a positive perception of PSC, and employees with 1-5 years of work experience were 0.15 (OR=0.85: p < 0.05) times less likely to have a positive perception of PSC. The intensive care staff was 1.29 (OR=1.29: p < 0.05) times more likely to perceive PSC positively. The general hospital staff was 2.88 times more likely to have a positive PSC score than specialty hospital staff (OR=2.88, p < 0.05).

**Table 8:** Factors influencing patient safety culture among HMC staff ^a^OR: odds ratio, ^ b^95% confidence interval of odds ratio, ​​​​​​​^c^P-value (level of significance < 0.05)

Variables	OR^a^	95%CI^b^	P-value^c^
Patient safety cultures dimension			
Teamwork Within Units	1.10	[1.05, 1.43]	<0.005
Supervisor/Manager Expectations & Actions Promoting Patient Safety	1.09	[1.04, 1.21]	<0.005
Organizational Learning—Continuous Improvement	1.43	[1.04, 2.35]	<0.005
Management Support for Patient Safety	1.04	[1.03, 1.14]	<0.005
Overall Perceptions of Patient Safety	1.01	[1.00, 1.11]	<0.005
Feedback & Communication About Error	1.05	[1.04, 1.19]	<0.005
Communication Openness	1.03	[1.02, 1.17]	<0.005
Frequency of Events Reported	1.08	[1.06, 1.20]	<0.005
Teamwork Across Units	1.05	[1.02, 1.19]	<0.005
Staffing	1.01	[1.00, 1.08]	<0.005
Handoffs & Transitions	1.04	[1.02, 1.13]	<0.005
Non-punitive Response to Errors	1.02	[1.00, 1.05]	<0.005
Gender (reference: female)			
Male	1.17	[1.03, 1.42]	<0.005
Age (years) (reference: 55 and above)			
Under 40	1.68	[1.25, 2.27]	<0.005
40 -55	0.74	[0.52, 1.06]	0.072
Position/profession (reference: others)			
HMC Administration	0.87	[0.67, 1.08]	0.211
Ambulance Paramedic	0.92	[0.43, 0.97]	<0.05
Staff Physician	1.04	[0.87, 1.44]	0.183
Pharmacist	0.89	[0.56, 1.18]	0.924
Physical, Occupational, or Speech Therapist	1.01	[0.82, 1.39]	0.418
Registered Nurses and midwives	1.14	[0.92, 1.58]	0.786
Technician (e.g., EKG, Lab, Radiology)	1.02	[0.86, 1.41]	0.572
Interaction with Patient (reference: I have direct contact with patients)			
I typically do not have direct interaction or contact with patients	1.16	[1.06, 1.65]	<0.005
Work Area (reference: emergency department)			
Intensive care unit (any type)	1.29	[1.10, 1.86]	<0.001
Laboratory and Radiology	0.92	[0.66, 1.27]	0.147
Medicine (non-surgical)	1.38	[0.89, 1.86]	0.063
Obstetrics	0.63	[0.58, 1.07]	0.427
Out-patient department	0.99	[0.69, 1.36]	0.072
Pediatrics	1.16	[0.93, 1.47]	0.566
Pharmacy	0.55	[0.49, 1.08]	0.054
Psychiatry/mental health	0.73	[0.65, 1.13]	0.829
Rehabilitation (including physical therapy)	0.31	[0.31, 1.01]	0.951
Surgery	1.97	[0.85, 2.98]	0.169
Work Experience (reference: less than one year)			
1 to 5 Years	0.85	[0.32. 0.97]	<0.005
6 to 10 Years	0.92	[0.83, 1.15]	0.861
11 to 15 Years	1.17	[0.97, 1.59]	0.625
16 Years or More	0.63	[0.41, 1.12]	0.794
Tenure with Current Specialty or Profession (reference: less than one year)			
1 to 5 Years	0.93	[0.83, 1.40]	0.422
6 to 10 Years	0.92	[0.73,1. 29]	0.647
11 to 15 Years	1.38	[1.06, 2.97]	<0.01
16 Years or More	0.63	[0.43, 1.11]	0.325
Hours Worked Per Week (reference:<40 hours per week)			
40 to 59 hours per week	1.21	[0.69, 1.66]	0.182
60 hours per week or More	0.85	[0.51, 1.27]	0.083
Types of Facilities (reference: Corporate departments)			
General Medical & Surgical Hospitals	2.88	[1.12, 4.24]	<0.001
Specialty Hospitals	1.24	[0.71, 1.73]	0.098
Clinical Support services	0.85	[0.43, 1.28]	0.214

## Discussion

HMC is a not-for-profit medical services company headquartered in Doha-Qatar. HMC services provide the best possible care for Qatar State residents. HMC employs more than 25,000 people from different countries with different understandings and perceptions of PSC. Since its start in 1979 until the time of the study, HMC has been operating nine hospitals, three community hospitals, ambulance services, a home care service, an ambulatory care center, and corporate departments [20]. There was a study conducted in Qatar for the Ph.D. degree. The study was done 13 years ago, including 800 nurses in eight departments of HMC facilities to assess their understanding of PSC [21]. the study had an approximately 60% response rate. The Agency for Healthcare Research and Quality's (AHRQ) Baseline Survey in US Hospitals was used to compare the results of the above-mentioned study. The comparison led to an in-depth assessment of the subscales' benefits and opportunities for improvement, and the results were comparable to US hospital performance assessments. The study concluded that the teamwork within the units was the main strength, and the nonpunitive response to errors was the lowest, indicating areas for improvement. within the units. Due to the small number of the participants and the participants were only nurses, and it was not multidisciplinary. Therefore, we decided to perform the current study with a bigger number of participants in different multidisciplinary HMC facilities.

The present study is instructive for a substantial number of employees (5,583) working in 17 HMC facilities in Qatar who participated in the survey. Furthermore, the workforce comprises individuals of varying ages, levels of expertise, and cultural backgrounds. Additionally, different professions (physicians, nurses, technicians, administrators, medical personnel), and different places of work (specialized hospitals, general hospitals, and corporate administration departments) were included in the study. All these elements have expanded the scope and significance of the present study's findings.

The mean response percentage of the targeted participants was 85.0%. Although the PSC concept is relatively new in HMC, in the present study, 10 HMC healthcare facilities responded with a rate of 100%, while the other HMC facilities responded with rates ranging from 71.4% to 97.5%, indicating that HMC employees are passionately committed to patient safety. The response rate differs between the professionals. In the present study, nurses had a higher response rate than others [22-24]. Furthermore, our results showed that employees who worked longer in the same specialties had a higher PSC response rate, supporting the previous report by Rajalatchumi et al. [25].

The overall mean PPR for the 12 dimensions of the PSC was 62.4%, ranging between 61.5% and 76.4%. It was considered moderate, and it could be improved. Mansouri et al. reported that in the Arab PSC review, Oman had 54.15% positive response rates, Egypt had 40.2%, Saudi Arabia had 56.7%, 42.3% in Jordan, and 54.3% in Kuwait [26]. Our overall mean PSC score was lower than the reported score in the USA (68.50%) [27], although it seems not significantly different. Despite similarities, differences were noted, which might be due to employment, construction programs, and different learning environments. Furthermore, factors such as more urbanized cities with better hospitals, better transportation, and enough professionals could affect the response rate. Other factors that made the differences PPRs were hospital type, size, and function [28].

The present study reported that three PSC dimensions (“teamwork between units,” “organizational learning and continuous development dimension,” and “patient safety management support”) had a mean PPR ≥ 75.0%. The “teamwork between units” dimension had the highest mean PPR (83.1%). This result agrees with studies from Lebanon and Jordan [29,30]. The second-highest mean PPR was 82.0% of the organizational learning, and continuous development dimension, which agrees with previous studies reported results [31-33]. PPR for the patient safety management support dimension was 79.2%, in agreement with Iran [34], Jordan [35], and Portugal [36] studies. The “feedback and communication about errors,” “teamwork across units,” “frequency of events reported,” “supervisory expectations and actions that promote patient safety,” and “overall perceptions of patient safety” dimensions had PPR scores between 50% and 70%. According to our findings, some discrepancies exist between the six dimensions regarding response rates that need to be addressed openly [27].

According to the results of this study, nonpunitive error response, communication openness, and staffing dimensions had positive response scores of 50.0%. These scores were considered weak and areas that required attention.

The nonpunitive error response dimension score of 27.7% represented an extremely low PPR, which might affect the overall mean PPR. However, the HMC authorities look for error incidents to improve services rather than blaming or retaliations; still, some employees are afraid to report the incidents. In various countries, such as China [37], Turkey [38], and Saudi Arabia [39], it has been demonstrated that hospitals are not exempt from criticism. Employees have expressed concerns that disclosing confidential information could lead to retaliation or termination. In this regard, they are creating an environment where reporting system failures rather than personal errors are essential for encouraging employees to report error events without fear of retaliation.

In the present study, low PPR scores in the staffing dimension show weakness in the overall PSC perception. This can lead to inadequate patient care, negatively affecting patient health. Additionally, a staff shortage can put pressure on staff, making staff members difficult to create a safe environment for patients. Hence, an adequate staffing ratio and enough team members are essential to ensure patient safety and good service [40,41].

In this study, the positive scores for open communication were low (46.6%), while higher scores were reported in the United States, Norway, and the Netherlands [42,43]. Several factors could contribute to the difference, including PSC perceptions, employees' understanding of PSC in their countries, years of experience, age, gender, and staff attitudes.

In the present study, 81.0% of participants graded PSC as good or excellent, similar to other studies conducted in the Gulf region that reported 74.5% to 83.5% [44]. Hence, the HMC staff's understanding of the PSC is comparable to or might be superior to neighboring countries.

Factors such as male gender, years of specialty experience, and hospital type significantly affected this study's HOSPSC perceptions mean score. Therefore, these factors should always be considered if PSC standards need to be improved [45].

Pearson correlation analysis for the 12 dimensions revealed a significant statistical correlation between seven HOSPSC dimensions and the PSC score's perception. The Pearson analysis of the other five PSC dimensions (“organizational learning and continuous improvement,” “open communication,” “nonpunitive response to errors,” “transfers and transitions,” and “overall perception of patient safety”) revealed no significant correlation. Furthermore, the study found a moderate correlation between “cross-unit teamwork” and “Frequency of Events Reported” and PSC scores perception because the Pearson correlation coefficient was 0.43 and 0.40, respectively. The last two dimensions should be carefully considered by hospitals committed to PSC because they have appropriate Pearson correlation coefficients compared to the other dimensions where PSC levels are high. Therefore, the instrument will be more efficient, and the PSC will be perceived as more positive [38].

The present study revealed factors associated with a decreased perception of PSC, including employees with 1-5 years of work experience and ambulance service employees. This decline of PSC perception among this HMC employees group might be due to the working environment, driving potential risks, short work experiences, and the extension of working time > 60 hours per day (for those who work overtime). Although the decline in PSC perception was reported previously [46], it is very important to explore this issue more in HMC and find solutions through further root-analysis studies and staff education programs.

According to our study, healthcare workers with over 15 years of experience in the work specialty have a more positive perception of PSC. The higher positive perception response rate is mostly due to the experienced staff members being more knowledgeable and familiar with the applications. Hence, experienced staff positively influence the PSC within the organization.

In this study, 12 dimensions were found that significantly predict the perception of PSC. However, the odd ratio values for the HSOPSC dimensions ranged from 1.01 to 1.10, which is a very narrow range and does not represent an accurate overall positive PSC perception prediction [47].

Our results showed that critical care professionals perceive PSC as more positive than other workplaces. However, clinical staff must collaborate to ensure that their expertise and knowledge apply to the best possible treatment for the patient. It ensures that adverse effects on their employees' health and well-being are avoided. Additionally, we found that healthcare professionals who have been in the same workplace for over five years are better equipped to promote a positive PSC experience and safeguard against detrimental factors.

Although the PSC establishment within HMC is good compared to the previous report [21], it still needs further improvement to adopt safe practices. Adoption of safe practices could be accomplished successfully when the healthcare professionals possess the skills and resources [46] and educate the staff to report the events early for prevention and better practice, and not for punishment. Furthermore, employee motivation, establishing a safety culture, encouraging employee contributions, and implementing best practices are necessary to establish a PSC environment. Moreover, PSC improvement requires the establishment of a dialogue concept between the patients and the staff [3]. We recommend that organizations should maintain a culture of safety awareness and vigilance to achieve successful patient safety initiatives. The staff members must comply with occupational health and safety regulations. As soon as management has provided the resources, they should be able to establish open and consistent communications with patients and solicit their feedback to improve the PSC concept. Due to the complexity, interconnectedness, and ever-changing nature of healthcare systems, a structured approach is necessary to meet educational needs. Although each employee has a job description and responsibilities, they must share information that helps to improve the PSC perception and concept [48]. Although the study had been conducted in different HMC facilities, the Qatar health system also includes the private sector and other facilities that are under the ministry of health but not under HMC cover. These issues might be considered limitations for the present study; however, we think they do not represent major issues regarding the PSC's understanding because the employees' characters are almost all similar.

## Conclusions

Staff understanding of the PSC and its perception of HMC facilities is moderate. However, more educational sessions and courses for all professionals must be implemented to better understand PSC among HMC employees. Furthermore, the trends of reporting errors and adverse events are not up to the standards among HMC employees; more efforts are needed to encourage employees to report errors in hospitals. In the present study, different medical and nonmedical professionals were involved in establishing the PSC concept's validity in HMC employees, making it possible to generalize it to all HMC facilities, other health sectors in Qatar, the Middle East Region, and even worldwide. Furthermore, this study can be used as a backbone for PSC understanding.
